# Identification and Expression Analysis of Long Noncoding RNAs in Fat-Tail of Sheep Breeds

**DOI:** 10.1534/g3.118.201014

**Published:** 2019-02-20

**Authors:** Mohammad Reza Bakhtiarizadeh, Seyed Alireza Salami

**Affiliations:** * Department of Animal and Poultry Science, College of Aburaihan; † Department of Biotechnology, University of Tehran, Tehran, Iran

**Keywords:** lncRNAs, Synteny, Gene regulatory network, QTL

## Abstract

Emerging evidence suggests that long non-coding RNAs (lncRNAs) participate in the regulation of a diverse range of biological processes. However, most studies have been focused on a few established model organisms and little is known about lncRNAs in fat-tail development in sheep. Here, the first profile of lncRNA in sheep fat-tail along with their possible roles in fat deposition were investigated, based on a comparative transcriptome analysis between fat-tailed (Lori-Bakhtiari) and thin-tailed (Zel) Iranian sheep breeds. Among all identified lncRNAs candidates, 358 and 66 transcripts were considered novel intergenic (lincRNAs) and novel intronic (ilncRNAs) corresponding to 302 and 58 gene loci, respectively. Our results indicated that a low percentage of the novel lncRNAs were conserved. Also, synteny analysis identified 168 novel lincRNAs with the same syntenic region in human, bovine and chicken. Only seven lncRNAs were identified as differentially expressed genes between fat and thin tailed breeds. Q-RT-PCR results were consistent with the RNA-Seq data and validated the findings. Target prediction analysis revealed that the novel lncRNAs may act in cis or trans and regulate the expression of genes that are involved in the lipid metabolism. A gene regulatory network including lncRNA-mRNA interactions were constructed and three significant modules were found, with genes relevant to lipid metabolism, insulin and calcium signaling pathway. Moreover, integrated analysis with AnimalQTLdb database further suggested six lincRNAs and one ilncRNAs as candidates of sheep fat-tail development. Our results highlighted the putative contributions of lncRNAs in regulating expression of genes associated with fat-tail development in sheep.

Sheep are a major source of meat and agricultural products worldwide. Fat content and deposition in sheep varies and is dependent on breed. In fat-tailed sheep breeds, efficiency of fat deposition in the tail is remarkably higher than that in other parts of the carcass, such as longissimus dorsi muscle and subcutaneous adipose (Bakhtiarizadeh *et al.* 2013). Fat-tail is developed as a survival mechanism for animals in hazardous environments and is a valuable energy reserve for the animal during migration and drought food deprivation. However, in todays’ modernized production systems, there has been a tendency among farmers to raise their sheep in more intensive and semi-intensive systems of production. On the other hand, as more energy is required to deposit fat in the body or tail than for accretion of an equivalent amount of lean tissue, producers are interested to reduce the costs of fat deposition by shifting the nutrient partitioning toward leaner carcass production (Vatankhah *et al.* 2006; Moradi *et al.* 2012). This has also made the decrease in fat-tail size, an interesting breeding purpose for sheep breeders. There are over than 28 indigenous sheep breeds in Iran, all of which except of Zel breed have large fat-tails (Valizadeh and Box 2010). Among, Lori-Bakhtiari is a typical fat-tailed breed in the southwestern part of Iran (the Zagros Mountains) having the largest fat-tail by girth and weight among all Iranian sheep breeds (Vatankhah *et al.* 2016). In contrast, Zel is the only thin-tail Iranian breed and is small in size, which is largely restricted to the northern slopes of the Alburz mountain near the Caspian Sea. Significant differences in fat deposition make these two breeds an ideal model to investigate the regulatory mechanism of fat-tail development (Vatankhah *et al.* 2006; Bakhtiarizadeh *et al.* 2013). Toward better understanding the regulatory mechanism of fat-tail development, comparative genomics of these breeds may help us identify a set of core molecular mechanisms through which fat tail formation may occur and thereby assisting breeders in the design of new breeding strategies to modulate fat deposition. For instance, in a previous study, we showed that FABP4 can be considered as an important candidate gene associated with fat-tail development in Lori–Bakhtiari breed (Bakhtiarizadeh *et al.* 2013). Moreover, a few gene expression-based studies have been performed on different sheep breeds to identify the crucial genes and/or molecular pathways involved in fat-tail development. A study on a fat-tailed (Kazak sheep) *vs.* a short-tailed breed (Tibetan sheep) led to identification of 646 differentially expressed genes (DEGs), including 280 up-regulated and 366 down-regulated genes among them NELL1 and FMO3 showed the largest fold change (Wang *et al.* 2014). Also, differential gene expression analysis of fat-tail in Han and Dorset sheep breeds, with extreme phenotypes for fat-tail content, revealed 602 DEGs that were involved in pathways related to lipid metabolism, of which the most significant one was triglyceride biosynthetic process (Miao *et al.* 2015b). In a more recent study 54 differentially expressed miRNAs were identified in Han and Dorset sheep breeds by small RNA deep sequencing technology. Functional enrichment analysis of the predicted target genes of these miRNAs demonstrated a less active lipid metabolism in adipose tissue of the Han sheep (Miao *et al.* 2015a).

As mentioned, gene expression analysis of mRNA and miRNA have been conducted in previous studies to compare the transcriptome profile of fat-tail between different sheep breeds. Nevertheless, the molecular genetics mechanisms underlying the fat-tail phenotype and fat deposition in fat-tailed sheep remain to be characterized on a genome-wide level. On the other hand, recent studies have shown that non-coding RNAs (ncRNAs) such as long ncRNAs (lncRNAs) can be considered as critical regulators involved in mammalian development (Iyer *et al.* 2015). The lncRNAs are generally defined as RNA molecules larger than 200 bp in length, which barely have protein-coding potential (Bakhtiarizadeh *et al.* 2016; Quinn and Chang 2016). They are transcribed and processed in a manner similar to mRNAs including transcription by RNA polymerase II, presence of 5′-cap and 3′-poly (A) and splicing (Wu *et al.* 2017). In contrast to mRNAs, they are less abundant, are not well conserved among species and have higher tissue specificity (Bakhtiarizadeh *et al.* 2016). Based on their genomic structural characteristics and their location relative to the nearest protein-coding genes, lncRNAs can be divided into at least four groups including, 1) intergenic (lincRNAs), 2) intronic (ilncRNAs), 3) bidirectional (share promoters with protein-coding genes) and 4) sense and antisense lncRNAs (Quinn and Chang 2016). With the tremendous progress of high-throughput sequencing technologies (RNA-Seq approach) and bioinformatics methodology, it is possible to detect a large number of novel transcripts including lncRNAs (Bakhtiarizadeh *et al.* 2016; Weikard *et al.* 2017). In our previous study, the first list of lincRNAs (n = 325) in eight different tissues of sheep was reported (Bakhtiarizadeh *et al.* 2016). In a more recent study, 11,646 novel lncRNAs were identified in 11 sheep tissues (Bush *et al.* 2018). Nonetheless, when compared to other mammals (such as human, mouse, cattle and pig), fewer lncRNAs have been annotated in the sheep genome (Weikard *et al.* 2017), necessitating that similar studies on sheep are performed. Moreover, emerging studies have shown that lncRNAs play key roles in regulating diverse biological processes (reviewed in (Weikard *et al.* 2017; Kopp and Mendell 2018)), specially lipid homeostasis (Bakhtiarizadeh *et al.* 2016; Chen 2016; Weikard *et al.* 2017). In this context, regulatory functions of lncRNAs in lipid metabolism have been already demonstrated in different organisms, including human (Chen 2016; Gao *et al.* 2018), mouse (Sun *et al.* 2013a), pig (Palmieri 2014; Zhou *et al.* 2015), bovine (Zhou *et al.* 2014) and chicken (Muret *et al.* 2017). There are a few transcriptome studies with a focus on the investigation of potential regulatory roles of lncRNAs in specific sheep tissues using RNA-seq, such as skeletal muscle (Li *et al.* 2018), skin (Yue *et al.* 2016), ovaries (Miao *et al.* 2016a, 2016b) and testis (Zhang *et al.* 2017). These studies reinforce that lncRNAs are widely involved during sheep development, like other mammals. However, no comprehensive study to date, has investigated regulatory functions of lncRNAs in fat deposition in sheep and related molecular pathways underlying fat-tail development. Therefore, to better understanding of the molecular mechanisms that might regulate fat-tail development and to reduce the fat content in tail, characterizing the lncRNAs and investigating how they might regulate the expression of mRNAs enable us to identify candidate lncRNAs that might be driving fat deposition. This can also help us to promote genetic improvement in sheep herds. In the current study, RNA-Seq data along with a computational approach was employed to understand the possible roles of lncRNA during fat-tail development in the two Iranian sheep breeds. Our main goals were: 1) Search for DEGs (lncRNAs and mRNAs) that may affect fat deposition in sheep by comparing the transcriptomic profiles of these breeds and 2) Discovery of the novel lncRNAs. Our findings expand the available catalog of lncRNAs in the sheep genome and will help to further understand the function of lncRNAs in fat deposition in sheep.

## Materials And Methods

### Ethics statement

All experimental procedures involving sheep breeds in this project were reviewed and approved by the research council of the University of Tehran.

### Sample collection

The adipose tissue samples were collected under sterile conditions from fat-tail of six male lambs (three Lori-Bakhtiari and three Zel sheep breeds). These lambs were weaned at age of 90 days, on average, and then reared under the same environmental conditions at the research station of the college of Aburaihan, University of Tehran (Ghezlagh farm). Animals were housed in individual pens on the same nutritional conditions with *ad libitum* access to the same diet and water, for 120 days. At the age of seven months, the animals were slaughtered and the fat-tail tissue samples (fat tissues of last lumbar vertebrae of tail) were immediately stored in liquid nitrogen and then were kept at -80° until total RNA extraction.

### RNA-Seq and quality control

Tripure Isolation Reagent (Roche Applied Science) was used to extract total RNA from samples according to the standard protocol with small modifications. Fat tissue was powdered using liquid nitrogen, but not to a fine powder. After homogenization, the homogenate was centrifuged at 12,000 × g for 10 min at 4° to remove the insoluble material. The layer of fatty material on the surface of the aqueous phase was removed carefully and the clear supernatant was proceeding with purification steps. The quantity of total RNAs were checked using NanoDrop (Thermo Scientific NanoDrop 2000), and 28S/18S ratio (28S and 18S ribosomal RNA bands) was evaluated by electrophoresis on 1% agarose gel to monitor the RNA integrity and contamination. RNA samples with an OD260 nm/OD280 nm ratio greater than 1.9 and a 28s/18s ratio greater than 1.8 were selected for RNA-Seq. Also, Bioanalyzer 2100 (Agilent, Santa Clara, CA) was employed to measure RNA integrity number (RIN) and RNA samples with a RIN greater than 7 were considered for cDNA library preparation and later RNA-sequencing. RNA purification, cDNA library construction (based on poly-A capturing method) and RNA sequencing were conducted by BGI company. Each library was paired-end sequenced (2 × 150 bp) on an Illumina HiSeq2000 platform. The raw sequencing reads were subjected to quality control using FastQC (v0.11.5) (Andrews 2017). Low-quality reads/bases and adaptor sequences were trimmed by Trimmomatic software (v0.35) (Bolger *et al.* 2014), which was set to keep reads longer than 120 bp with a minimum phred score of 20. After trimming, the remaining clean reads were subjected to downstream bioinformatics analyses.

### lncRNA identification pipeline

**Figure 1:**
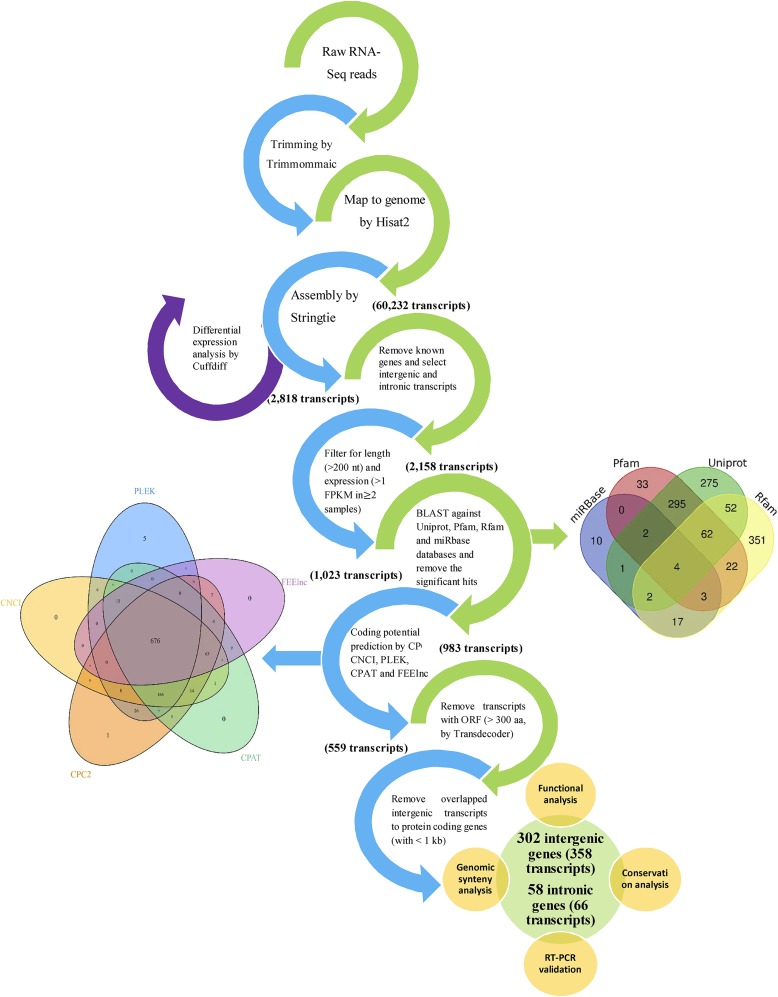
The filtering pipeline for identification and annotation of novel potentially functional lncRNAs in sheep. Venn diagrams show the results of five coding potential prediction tools (left venn diagram) and balst against four different databases (right venn diagram).

The stringent stepwise filtering pipeline was used to detect the lncRNAs (Figure 1). First, the clean reads were mapped to sheep genome (Oar_v3.1) using Hisat2 program (version 2.1.0) (Kim *et al.* 2015). Then, the transcriptome was assembled by Stringtie software (v1.3.4d) based on the Ensembl sheep reference annotation GTF file (release 88) (Pertea *et al.* 2015). All assemblies were merged into a reference transcriptome to generate a unique set of all transcripts, using Stringtie software. The expression values of all identified transcripts were calculated as FPKM (fragments per kilobase per million) and Cuffdiff tool (v2.2.1) (Trapnell *et al.* 2010) was used to identify DEGs between two sheep breeds at both transcript and gene level using a beta negative binomial model. Differential expression analysis was run with FPKM upper-quartile normalization (to improve the strength of the differential expression calls for less abundant transcripts) and multiple read corrections. Transcripts with a false discovery rate (FDR) ≤0.1 were considered as DEGs. In the next step, Cuffcompare (v2.2.1) (Trapnell *et al.* 2010) was used to identify transcripts in the “i” (intronic transcripts) and “u” (intergenic transcripts) classes and detected transcripts that overlap with annotated genes, based on the reference transcriptome. To do this, assembled transcripts were compared to Ensembl sheep reference annotation GTF file (release 88). All “gene_biotype=lncRNA” groups in the GTF file were considered as annotated lncRNAs. The remaining transcripts with at least one exon and longer than 200 nt were retained for further analysis and a comprehensive and stringent filtering pipeline was applied to distinguish lncRNAs from all the assembled transcripts, as follow:

1. The transcripts with one exon and longer than 10000 nt were excluded.
2. The transcripts with one exon overlapping simple repeats were removed (based on UCSC RepeatMasker file).
3. The transcripts with FPKM ≥ 1 that were expressed in at least two samples were kept.
4. The transcripts with at least one significant (E-value < 1e-5) hit against UniprotKB (by BLASTx), miRbase (release 21, by BLASTn) and Rfam (by BLASTn) databases were excluded.
5. The transcripts with any known protein domains documented in the Pfam database were filtered out, using hmmscan from the HMMER 3.1b2 package.
6. Five coding potential prediction tools with different intrinsic sequence-related features (composition, structural properties and motifs) and divergent filtering steps (Weikard *et al.* 2017) including CPC2 (score > 0.5) (Kang *et al.* 2017), CNCI (score > 0) (Sun *et al.* 2013b), CPAT (score > 0.36) (Wang *et al.* 2013), PLEK (score > 0) (Li *et al.* 2014) and FEElnc (default parameters) (Wucher *et al.* 2017) were employed and the transcripts that were predicted as protein coding genes by at least three of above tools were removed.
7. The open reading frame (ORF) predicted using TransDecoder tool (v2.1.0) (https://transdecoder.github.io/) and the transcripts with an ORF <300 aa were considered for the next step of the analysis.
8. The intergenic transcripts that were located in a distance < 1 kb to a known protein-coding gene were excluded.

Finally, the set of remaining transcripts were considered as candidate lncRNAs and classified into two groups including lincRNAs (occurred between mRNA genes without any overlap) and ilncRNAs (located entirely within an intron of a mRNA gene). Also, the genomic positions of candidate lncRNA were compared against lncRNAs positions reported in our previous study (Bakhtiarizadeh *et al.* 2016) and (Bush *et al.* 2018). To investigate the breed-specific lncRNAs, we considered one lncRNAs as breed-specific, if that lncRNA was expressed in at least two samples of one breed and was not expressed in all the samples of the other breed.

### Target prediction of the candidate lncRNAs and PPI network

It is well known that lncRNAs, as a kind of noncoding RNAs, can regulate the expression of the genes in a host (in case of ilncRNAs) (Petazzi *et al.* 2013) or nearest neighboring mRNAs (in case of lincRNAs). This is possible through the transcriptional activation/repression or epigenetic modification, which refers to cis regulation (Weikard *et al.* 2017; Kopp and Mendell 2018). Accordingly, potential target genes of the lincRNAs were identified by searching the protein coding genes located 100 kb upstream and downstream of each lincRNA and nearest genes were considered as *cis*-regulated genes. On the other hand, trans role refers to lncRNAs that regulate other genes on different positions of the genome, which can be identified based on expression analysis and finding co-expressed mRNAs with candidate lncRNAs. Therefore, to identify the target genes that were potentially regulated by lncRNAs *in trans*, the expression levels of the candidate lncRNAs and the known mRNAs were used and Pearson’s correlation coefficient between each pair of lncRNA and protein-coding gene was calculated (r > 0.99 or < -0.99 and *P* < 0.00005). Functional annotation of the candidate lncRNAs was performed based on the functional enrichment analysis of their related cis and trans target protein-coding genes.

Moreover, each of the trans target gene datasets (lincRNAs and ilncRNAs) were subjected to protein-protein interaction (PPI) network analysis using STRING database (v 10.5) to investigate these genes form interactive PPI networks. Then, significant PPI networks and co-expressed lncRNA-mRNAs (based on correlation) were integrated to construct the final network. ClusterONE plugin (Clustering with Overlapping Neighborhood Expansion, version 1.0) in Cytoscape was used to detect the modules (sub-networks) in the integrated network. A cut-off value of *P* ≤ 0.01 and the minimum number of genes in a cluster >5 were utilized to measure the significance of the predicted modules (Nepusz *et al.* 2012). Cytoscape software (version 3.6) was applied to visualize the integrated network (Shannon *et al.* 2003).

### Functional analysis

The Enrichr web server (Kuleshov *et al.* 2016) was used to perform functional enrichment analysis for the annotated DEGs, the potential targets of lncRNAs and the genes in identified modules based on gene ontology (GO, biological process) and KEGG pathway categories. Only terms with false discovery rate (FDR) < 0.05 were considered significant.

### Analysis of Conservation

BLASTn was used to evaluate conservation of the novel lncRNAs with human, bovine and chicken with an E-value<=1e-5 cut off. To do this, lncRNA sequences of these organisms were downloaded from the NONCODE database (v5.0). The same method was applied to compare sheep coding sequences against coding sequences of human, bovine and chicken. Protein coding sequences of these organisms were obtained from Ensembl database.

### Genomic synteny analysis

To assess the synteny of the candidate lincRNAs, nearest upstream and downstream protein-coding genes of the lincRNAs were compared to neighboring protein-coding genes of known lincRNAs in human, bovine and chicken. To this end, genomic positions of lincRNAs were extracted from the NONCODE database (v5.0) for above organisms and nearest upstream and downstream protein-coding genes were extracted and used to identify syntenic loci. Suppose, there is a novel lincRNA between X and Y protein coding gene. If we can find one lincRNA (maybe with different sequence) in human (for example) that locates between X and Y protein coding gene, this lincRNA can be considered as conserved syntenic gene.

### Novel lncRNAs and QTL analysis

To study if novel lncRNAs were located in quantitative trait loci (QTL) associated with lipid metabolism, a co-localization analysis of respected genes was performed. First, all the sheep QTL related to lipid metabolism were obtained from AnimalQTLdb (Hu *et al.* 2016). Then, the positions of the lincRNAs and ilncRNAs were compared to the positions of the QTL. The novel lncRNA genes with the start and end positions within the QTL regions were considered as successfully annotated in the QTL.

### Q-RT-PCR validation

Validation of nine lincRNA transcripts (randomly selected) was performed by using Quantitative Real-time PCR (Q-RT-PCR). Total RNA was extracted from fat-tail tissues of 10 samples (six samples from the same fat-tail tissues as that for RNA-Seq analysis and four new samples from two Lori-Bakhtiari and two Zel breeds), using Tripure Isolation Reagent (Roche Applied Science). cDNAs were synthesized using RevertAid First Strand cDNA Synthesis Kit (Thermo Fisher, Co., USA), according to the manufacturer’s instructions. Q-RT-PCR was performed using Light Cycler 96 instrument (Roche Co. Germany) using SYBR Green Master Mix (Thermo Fisher Scientific, USA). GAPDH housekeeping gene was used as an internal standard and relative gene expression levels were calculated using the comparative Ct method with the equation 2^-ΔΔCt^. To compare the results of Q-RT-PCR with the RNA-Seq results, the mean 2^−ΔΔCT^ value for each gene was transformed into a fold change. The primers used in these analyses were designed online using Primer3Plus software (Untergasser *et al.* 2007) and are listed in Supplementary File S1.

### Statistical analysis

The ANOVA procedure in R software was used for statistical analysis of different features among novel and annotated lncRNAs and mRNAs as well as relative expression of genes in different samples. Tukey’s multiple comparisons test (*P* < 0.05) was considered significant.

### Data availability

The raw RNA-Seq data were deposited in SRA database, with the BioProject accession number of PRJNA508203 (Release date: 2020-01-01). File I1 contains Figures I1, I2 and I3 related to hierarchical clustering analysis of all the biological samples, novel lncRNAs characteristics and gene expression patterns of different genes in different samples, respectively. File S1 contains primer sets used for Q-RT-PCR. File S2 contains different characteristics of the identified novel lincRNAs and ilncRNAs. File S3 contains results of blast novel lncRNAs against different species. File S4 contains results of syntenic analysis of novel lincRNAs against different species. File S5 contains results of co-expression analysis between novel lncRNAs and protein coding genes. File S6 contains detailed information related to the identified modules in integrated network. File S7 contains list of GO terms and KEGG pathways for different modules in integrated network. File S8 contains results of QTL analysis. Supplemental material available at Figshare: https://doi.org/10.25387/g3.7700972.

## Results

### RNA sequencing and mapping

To identify novel lncRNAs as well as potentially functional lncRNAs involved in fat-tail development, six cDNA libraries were sequenced using Illumina HiSeq2000 platform. Totally, 66.8 and 62,9 million raw paired-end reads were obtained in Lori-Bakhtiari and Zel sheep breeds, respectively. Pre-processing and low-quality trimming processes removed only 1,104 reads, indicated that the data were quite appropriate for subsequent analysis. On average, more than 85% of the clean reads were aligned to the sheep reference genome in each sample. Also, more than 79% of the clean reads were uniquely aligned in each sample (Table 1). A hierarchical clustering analysis was performed (based on gene expression values of all the expressed genes (as FPKM) and using cummerbund R package) to gain insight in to the relationship among different samples. Results showed that the samples were grouped correctly and two main clusters formed as a result of different sheep breeds (Supplementary Information I1, Figure I1).

**Table 1:** Summary of RNA-Seq data and mapping

Breeds	Raw reads	Trimmed reads	Concordantly mapped reads	Discordantly mapped reads	Uniquely mapped reads (%)	Total mapping
Lori-Bakhtiari_1	26,282,890	26,282,599	22,134,586	1,938,138	22,408,851 (0.85)	24,072,724 (0.92)
Lori-Bakhtiari_2	20,075,866	20,075,798	13,620,516	3,029,187	15,633,869 (0.78)	16,649,703 (0.83)
Lori-Bakhtiari_3	20,428,424	20,428,247	13,552,931	3,326,710	15,676,435 (0.77)	16,879,641 (0.83)
Zel_1	22,292,871	22,292,640	17,922,852	1,758,155	17,456,499 (0.78)	19,681,007 (0.83)
Zel_2	20,164,542	20,164,351	13,501,701	3,320,607	15,650,133 (0.78)	16,822,308 (0.83)
Zel_3	20,532,170	20,532,024	14,677,141	3,166,595	16,127,823 (0.79)	17,843,736 (0.87)

### Novel lncRNA identification

**Figure 2:**
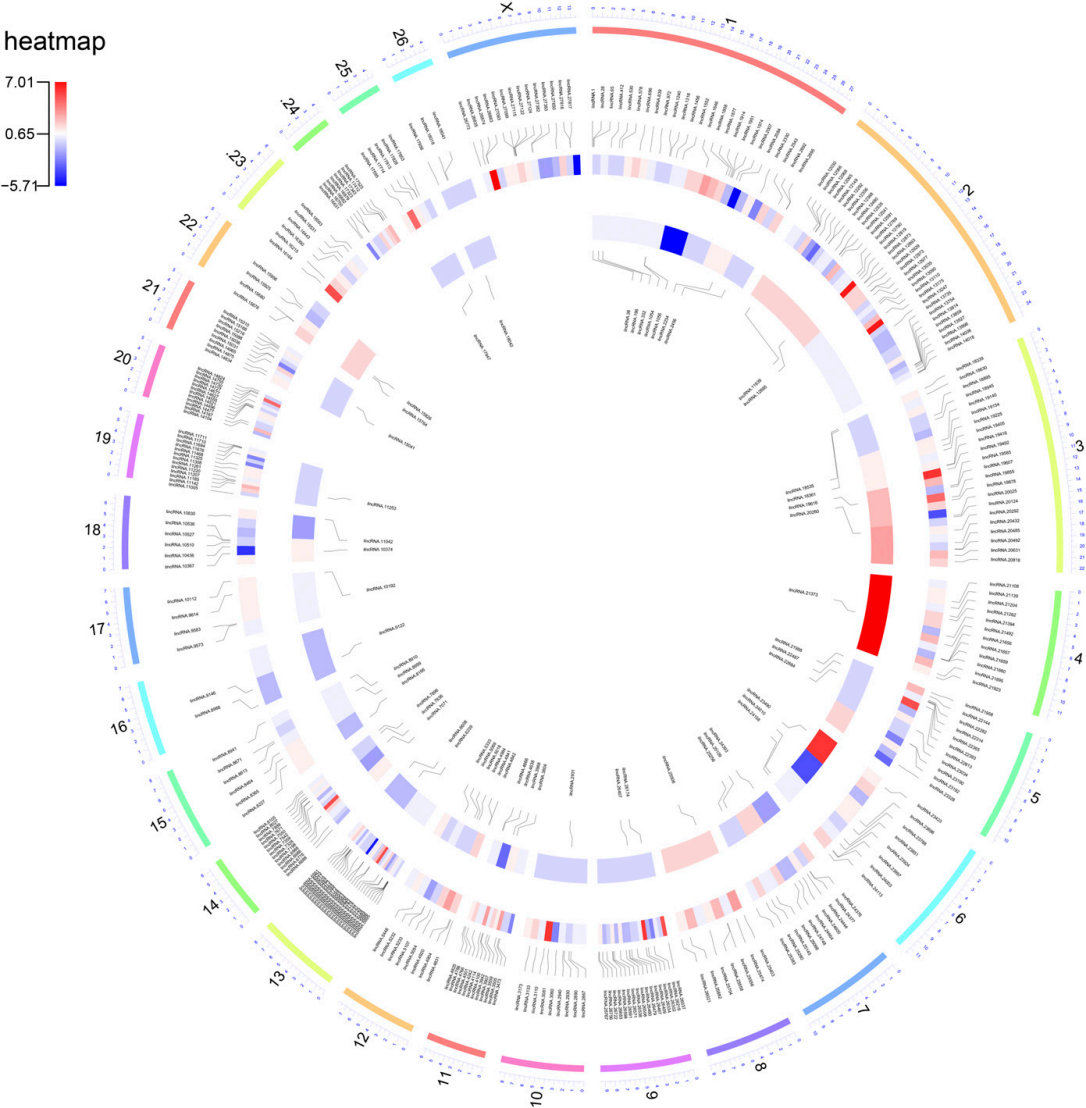
Circos plot represents the genome-wide distribution density of all the identified novel lncRNAs (in clockwise order). The outer ring of the plot shows the chromosomes, and the central and inner rings exhibit the log2 fold change (Lori-Bakhtiari against Zel) of the novel lincRNAs and ilncRNAs, respectively. Red heatmap colors indicate the higher expression of the gene in Lori-Bakhtiari than Zel breed. Also, the position of the novel lincRNAs and ilncRNAs are shown in the central and inner rings, respectively.

After reconstructing the transcriptome for each sample and combining all assemblies into a reference transcriptome through Stringtie software, a total of 60,232 transcripts were identified including 18,399 protein coding transcripts. Of which, 29,099 transcripts were known, including 815 known lincRNAs transcripts (242 genes). Of the remaining un-annotated transcripts, 2,399 transcripts were classified with class code ‘u’ (intergenic) and 419 transcripts were annotated as intronic (class code “I”). Using a rigorous filtering pipeline and removing tRNAs, rRNAs and other ncRNAs by blast search and also removing transcript with coding potential, 358 novel lincRNAs and 66 novel ilncRNAs transcripts corresponding to 302 and 58 gene loci were detected, respectively (Figure 1). These lncRNA transcripts were distributed throughout all sheep chromosomes (except the Y chromosome), although chromosome 2 contained the largest number of lncRNAs. The numbers of the novel lncRNAs distributed among the different chromosomes along with their expressions are shown in a Circos plot (Figure 2). Out of all identified novel lncRNAs, 16 lincRNAs and three ilncRNA genes were identified as breed-specific lncRNA genes (these genes are marked in Supplementary File S2). Out of 16 breed-specific lincRNAs, 10 and five lincRNAs were expressed in Lori-Bakhtiari and Zel sheep breeds, respectively. Also, out of three breed-specific ilncRNAs, two and one ilncRNA genes were expressed in Lori-Bakhtiari and Zel sheep breeds, respectively. It was an interesting result, as the number of breed-specific lncRNA genes in Lori-Bakhtiari (fat-tailed breed) was twice, which can be related to larger fat-tail in this breed. The complete information of the novel lncRNAs is provided in Supplementary File S2. Of the novel transcripts (358 novel lincRNAs and 66 novel ilncRNAs), 96 lncRNA transcripts were mapped to the known sheep lncRNAs, including 58 lincRNAs related to our previous study (Bakhtiarizadeh *et al.* 2016) and 69 transcripts (including 63 lincRNAs and six ilncRNAs) related to another previous study (Bush *et al.* 2018).

### lncRNAs features

Different features of the novel lncRNAs were compared against annotated lncRNAs and mRNAs, to evaluate their possible potential as new candidates (Supplementary Information I1, Figure I2). Since, all the annotated lncRNAs in sheep annotation file (Ensembl GTF file release 88) were lincRNAs, features of the novel ilncRNAs were compared against sheep annotated mRNAs. There were 22,841 protein-coding transcripts corresponding to 14,903 genes as well as 815 known lincRNA transcripts corresponding to 242 lincRNA genes in the sheep genome, based on Ensembl sheep genome annotation (release 88). The results showed that the average GC content of the novel and annotated lincRNAs were the same (0.48%), however novel ilncRNAs had higher GC content (51%) that was lower than known mRNAs (53%).

The size distribution of the novel lincRNAs transcripts ranged from 200 to 5,711 bp. The average length of these transcripts was 769 bp, which is shorter than that of the annotated lincRNA transcripts (2,453 bp) and protein-coding transcripts (2,022 bp). The range of the novel ilncRNAs transcripts were 202 to 3,914 bp, with an average length of 787 bp. The shorter length of the novel than annotated lincRNAs can be attributed to inclusion of transcripts with one exon into the analysis in this study.

Most novel lncRNAs had two exons (240 of 360 lncRNAs, 0.66%) and the average exon number of the novel lincRNA and ilncRNA genes were 2.62 and 2.50, which are similar to that of the annotated lincRNA genes (2.35), and significantly less than that of the known mRNAs (10.28). Over 71 and 59% of the novel ilncRNAs and lincRNAs had no more than 2 exons, while above 83% mRNAs contained no less than three exons.

All the lncRNAs tended to be expressed at a lower level than the mRNAs. The lower expression of lncRNAs than protein coding genes were reported in other studies (Arrial *et al.* 2009; Bakhtiarizadeh *et al.* 2016). The average expression value of annotated lincRNAs, novel lincRNAs, novel ilncRNAs and mRNAs in different samples were 2.6, 2.00, 2.00 and 3.1, respectively. Comparisons among different types of genes and different samples are provided in Supplementary Information I1, Figure I3. lncRNAs in all samples were expressed at similar levels.

### Analysis of Conservation

To explore how much lncRNAs are conserved and find the putative mammalian orthologs of these genes, BLASTn was applied to directly compare the novel lncRNAs with that in bovine, chicken and human. Only, 121 and 52 significant hits (E-value<=1e-5) were found in bidirectional comparisons between the novel lincRNAs with bovine and human, respectively. Totally, 152 novel conserved lincRNAs transcripts related to 125 genes were found, among them 34 genes were common in both species. Also, 15 and five novel ilncRNAs were evolutionary comparable with lncRNA sequences from bovine and human, respectively. Eighteen novel conserved ilncRNAs transcripts (14 genes) were found, which three of them had orthologs in both bovine and human. These findings confirm the previous reports that a small proportion of lncRNAs in vertebrates retains interspecies short and highly conserved regions (Ulitsky *et al.* 2011). Less than 39% (139 of 359) of the novel lncRNAs were conserved among the investigated species, which is in agreement with previous study in sheep (42%) (Bush *et al.* 2018). In this regard, the conservation proportion among lncRNAs in human and mouse ranged from 14 (Bajic *et al.* 2006) to 27% (Sasaki *et al.* 2007).

The longest conserved lincRNA sequence length was 2,299 nt (lincRNA.24664.4), which matched a lncRNA in human (NONHSAT234783.1). Also, the average alignment length between novel lincRNAs and bovine and human lncRNAs were 284 and 312 nt, respectively (Supplementary File S3). The longest conserved length of the novel ilncRNAs was identified between ilncRNA.1055.1 and NONBTAT018061.2 in bovine (1,236 nt). Moreover, the average alignment length between novel ilncRNAs and bovine and human lncRNAs were 436 and 234 nt, respectively (Supplementary File S3). On the other hand, screening the chicken lncRNAs identified no similar lncRNA sequences. To compare the alignment length of lncRNAs against alignment length of coding sequences, all sheep coding sequences were compared against bovine and human coding sequences. The results revealed that in protein coding sequences, long segments were typically very similar to their homologs as the average alignment length of sheep mRNAs against bovine and human genes were 1119 and 771 nt, respectively. However, these differences can be attributed to the greater length of lncRNAs compared to mRNAs.

### Synteny analysis

**Figure 3:**
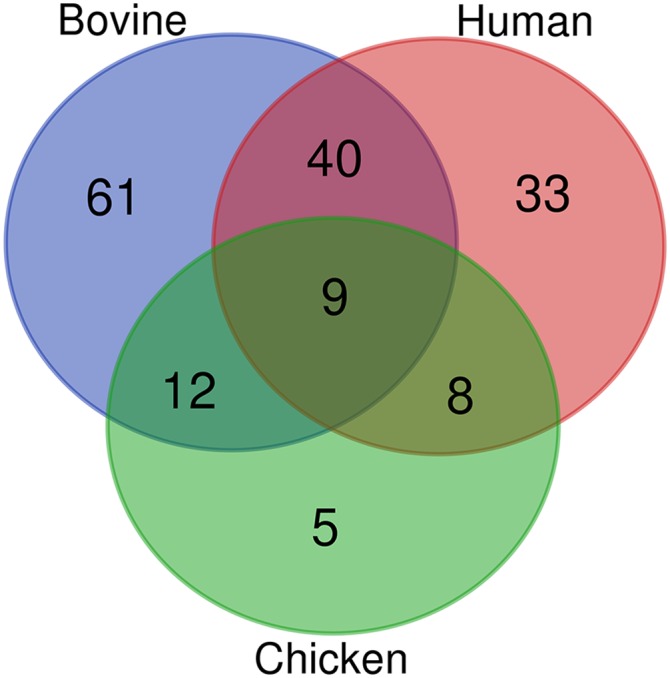
Venn diagram of the results of synteny analysis in different species. As can be seen, there are nine lincRNAs with conserved neighboring coding genes among sheep, bovine, human and chicken.

Inspecting genomic regions adjacent to the novel lincRNAs on sheep genome that are syntenic to the targeted bovine, human and chicken chromosomal regions, revealed a similar structural architecture. Through genome-wide synteny analysis, 122, 90 and 34 novel lincRNAs genes were found to be located in the neighborhood of respective orthologous protein coding genes in sheep/bovine, sheep/human and sheep/chicken comparisons, respectively (Supplementary File S4). Of 122 and 90 lincRNAs genes, 47 and 10 genes had conserved sequences in bovine and human, respectively. Totally, 168 novel lincRNAs genes showed conserved synteny with the investigated species. Of these, 70 genes showed evidence of sequence homology in bovine or human. Interestingly, nine and 60 novel lincRNAs genes showed the same neighboring coding genes within all species (sheep, bovine, human and chicken) or at least three species, respectively (Figure 3). Synteny analysis revealed that sheep and bovine had the largest numbers of lncRNAs with the same neighboring genes than the other investigated species, likely because they are closely related in evolution. In concordance with previous study (Necsulea *et al.* 2014), we also found that the number of synteny or conserved lncRNAs were reduced while we have a greater divergence among species. It is implied that lncRNAs with different evolutionary ages show various sequence constraint patterns.

### Differential expression analysis

To identify fat-tail related lncRNAs, differential expression was compared between two sheep breeds. The finding showed that mRNAs tended to be considerably differentially expressed than lncRNAs, as seven lncRNAs (including one up-regulated ilncRNAs, six up and one down-regulated lincRNAs) and 311 known mRNAs (including 215 up and 96 down regulated) were identified as DEGs. The lower number of significantly differentially expressed lncRNAs than mRNAs may have biological or technical reasons. Furthermore, among the eight detected lincRNAs, three and five genes were specifically expressed in Lori-Bakhtiari and Zel breed, respectively. Only one ilncRNA was identified as specifically expressed gene, and seen in Zel. These results suggesting that the specifically expressed lncRNAs may function in fat-tail development. The differentially expressed lncRNAs are shown in table 2.

**Table 2:** Differentially expressed lncRNAs and their cis target genes

lncRNA	Closest left mRNA	Closest right mRNA	Expression* in Lori-Bakhtiari	Expression* in Zel	FDR
lincRNA.27817	SLC25A53	FAM199X	0	5.13	0.008
lincRNA.26835	SHROOM2	CLCN4	12.82	0	0.008
lincRNA.25403	SMYD2	COL12A1	14.92	2.02	0.014
lincRNA.12819	ENSOARG00000015627	LGSN	47.27	0.47	0.035
lincRNA.16164	ENSOARG00000004542	TSHZ1	3.14	0	0.059
lincRNA.21492	CCM2	MYO1G	64.52	19.27	0.059
lincRNA.3473	ACACA	C17orf78	80.07	303.52	0.066
ilncRNA.20260	PRDM4	PRDM4	277.18	26.50	0.082

* Expression of genes are represented as FPKM.

### Q-RT-PCR validation

**Figure 4:**
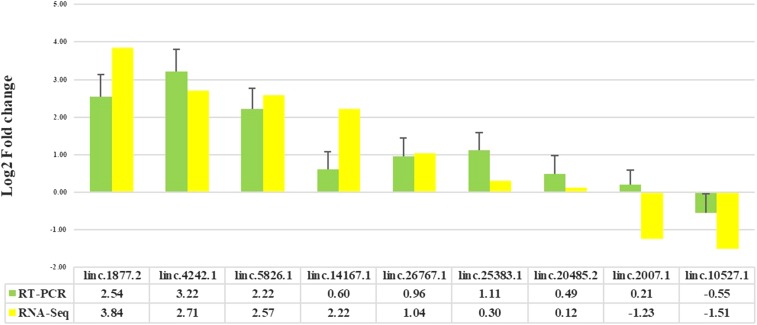
Q-RT-PCR validation of some of the novel lncRNA transcripts. Here, fold change indicates the ratio of average expression of Lori-Bakhtiari samples relative to that of Zel’s.

To verify the expression patterns of the lncRNAs determined by RNA sequencing, Q-RT-PCR was performed on nine randomly selected lincRNA transcripts. As shown in Figure 4, the findings showed that the expression patterns of eight lincRNA transcripts (except linc.2007.1) were consistent with the RNA-seq data, even though there were variations observed in these methods, owing to intrinsic features of the different approaches. These findings indicated that in most cases the Q-RT-PCR results were consistent with those of RNA-Seq, which implied that the identification pipeline is reproducible and reliable.

### Functional analysis

To assess the potential role of lincRNAs in regulatory processes, the *cis*-regulated genes of all the lincRNAs were searched using a threshold of 100 kb. All seven differentially expressed lincRNAs were detected close to 12 mRNAs (Supplementary file S2). Of which, four lincRNAs spaced less than 20 kb away from their putative mRNA targets. To assess the regulatory potential of the target genes, known transcription factors (TFs) in the sheep genome were extracted from AnimalTFDB3.0 database (Zhang *et al.* 2015) and compared to the target genes. The results showed that only one target gene (TSHZ1) is reported as TF. To better understand the roles of the identified lincRNAs and relevance in lipid metabolism, all target genes were subjected to GO and KEGG pathway analysis. The target genes were significantly enriched into 79 GO terms and nine KEGG pathways, which the most significantly enriched GO and KEGG pathway were “tryptophan catabolic process to acetyl-CoA” and “fatty acid biosynthesis”, respectively.

lncRNAs can also regulate expression of genes on other chromosomes or distal regions (*trans*-acting) (Jandura and Krause 2017). Hence, mRNAs that were co-expressed (based on correlation analysis > 0.95 or < -0.95) with lncRNAs were selected for functional analysis. A total of 572 interactions (including 568 positive and four negative correlations) were identified between 53 lincRNAs and 514 mRNAs. Of these, 23 lincRNAs had only one target gene each and 13 lincRNAs had more than five target genes each. Out of 514 target genes, 44 mRNAs were identified as TFs (based on AnimalTFDB3.0 (Zhang *et al.* 2015)) (Supplementary file S5). Results of functional enrichment analysis revealed only one KEGG pathway, “neuroactive ligand-receptor interaction”, which was significantly enriched in the target genes that were positively correlated with lincRNAs. Also, 14 GO terms and two KEGG pathways were significant in the target genes that were negatively correlated with lincRNAs including “cGMP-mediated signaling”, “receptor guanylyl cyclase signaling pathway”, “regulation of small GTPase mediated signal transduction” and “aldosterone-regulated sodium reabsorption”. Also, nine genes were found related to “calcium signaling pathway” including CHRM2, ADCY10, CALML5, HTR1E, GPR119, ATP2B2, ADCY1, SSTR1 and TSHR, which were regulated by lincRNA.13110, lincRNA.11185, lincRNA.1974, lincRNA.13110, lincRNA.13110, lincRNA.23034, lincRNA.13110, lincRNA.13110 and lincRNA.13110, respectively. Moreover, a total of 21 mRNAs and 10 ilncRNAs containing 21 relationships were detected. The target gene numbers of each ilncRNAs ranged from one to six. Out of 21 target genes, only one gene (CUX2) was identified as TF (based on AnimalTFDB3.0 (Zhang *et al.* 2015)) (Supplementary file S5). The prediction of ilncRNA functions was performed based on the functional enrichment analysis of their related trans target mRNAs and revealed that 32 GO terms were significant, containing “positive regulation of synaptic transmission”, “polyphosphate metabolic process”, “calcium ion transmembrane import into cytosol” and “chemical synaptic transmission”.

**Figure 5:**
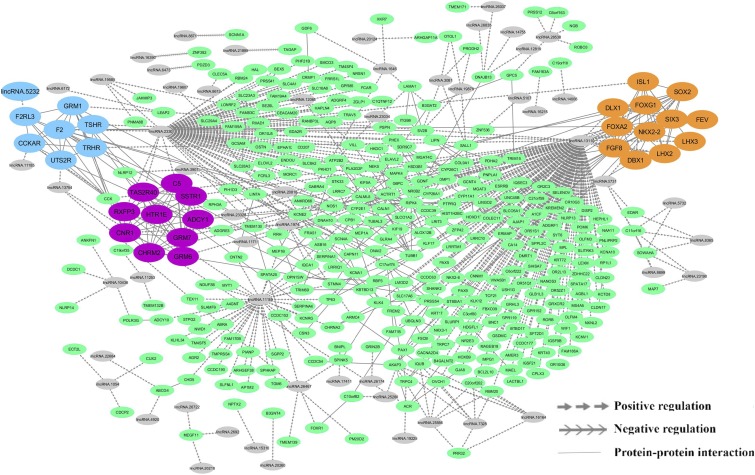
Integrated lncRNA-mRNA interaction network constructed based on co-expressed genes and STRING-derived PPI. Purple nodes: purple module (P-value = 0.00002); orange node: orange module (P-value = 0.0009); blue nodes: blue module (P-value = 0.02); gray nodes: lncRNAs; green nodes: protein coding genes.

Then, protein–protein interaction (PPI) analysis was performed using STRING database (v 10.5) (Szklarczyk *et al.* 2017) for the target gene datasets, separately. Only, one significant PPI network (p-value <1.0e-16) including 286 interactions and 330 genes was found among trans target genes that were positively correlated with lincRNAs. Next, PPI network and co-expressed lncRNA-mRNAs were merged, which comprised 55 lncRNAs and 351 protein-coding genes, including 676 interactions. ClusterONE tool was used to identify potential sub-networks. Four significant modules were found in integrated network and the size of these modules ranged from nine to 12. Since, most of the genes in the fourth module were overlapped with the second module, the most significant module (module two or orange module) was considered. The integrated network along with sub-networks is shown in Figure 5. Also, detailed information related to the modules are summarized in Supplementary file S6.

To assess whether the identified modules were biologically functional, enrichment analysis was performed for each module. Purple module was significantly enriched for 72 GO terms and nine KEGG pathways including “cellular response to lipid” and “cAMP signaling pathway”. Gene members of orange module were significantly enriched into 119 GO terms and two KEGG pathways, which were mainly associated with “regulation of insulin secretion”, “regulation of transcription”, “gene expression” and “neuron differentiation”. Enrichment analysis for blue module revealed that its members were enriched in 81 GO terms and 14 KEGG pathways including “regulation of lipid kinase activity”, “positive regulation of lipid metabolic process” and “regulation of lipolysis in adipocytes”, which are related to lipid metabolism. A comprehensive list of modules enrichment results is provided in Supplementary file S7.

### QTL analysis

To identify the potential lncRNAs involved in lipid metabolism, coincidence of the novel lncRNAs and QTL (related to lipid metabolism) was investigated. One hundred and forty-seven QTL representing 32 different phenotypic traits associated with lipid metabolism were retrieved from the sheep QTL database. Out of 301 lincRNAs and 58 ilncRNAs, 100 (on 13 different chromosomes) and 16 genes (on nine different chromosomes) were successfully located into 36 and 28 different QTL regions, respectively (further details in Supplementary file S8). Out of 100 lincRNAs, two lincRNAs (including lincRNA.16164 and lincRNA.12819) were DEGs. Also, 11 lincRNAs (including lincRNA.10436, lincRNA.12541, lincRNA.13110, lincRNA.16164, lincRNA.16215, lincRNA.1808, lincRNA.19585, lincRNA.1974, lincRNA.19878, lincRNA.22282 and lincRNA.3081) and one ilncRNA (ilncRNA.1054) were found as breed-specific. Interestingly, nine of 11 breed-specific lincRNA genes (including lincRNA.12541, lincRNA.13110, lincRNA.16164, lincRNA.16215, lincRNA.1808, lincRNA.19585, lincRNA.19878, lincRNA.22282 and lincRNA.3081) were expressed in Lori-Bakhtiari (fat tailed breed). This can be attributed to potential effect of these lincRNA genes in fat-tail development in this breed. Moreover, six lincRNAs (lincRNA.26756, lincRNA.17595, lincRNA.6399, lincRNA.2930, lincRNA.2940 and lincRNA.18895) and one ilncRNA (ilncRNA.2931) were located in QTL regions related to “Tail fat deposition” (Table 3). Owing to large intervals of sheep QTL regions, lncRNAs that were located in QTL< 1 Mb were further considered. Interestingly, only three lincRNAs were found that all of them were in QTL regions associated with “Tail fat deposition” (further details in Supplementary file S8).

**Table 3:** Identified novel lncRNAs in QTL regions related to “Tail fat deposition”

lncRNA	lncRNA position*	QTL id	QTL position*	Closest left mRNA	Closest right mRNA
lincRNA.26756	9: 93793209-93797530	127008	9: 93460494-93799321	DSCC1	—
lincRNA.17595	25: 7612489-7615461	127015	25:202582-7694641	—	TOMM20
lincRNA.6399	13: 63356541-63371778	127012	13: 62767123-63461553	—	ITCH
lincRNA.2930	10: 28841431-28846038	126989	10: 26820350-32294189	—	N4BP2L1
lincRNA.2940	10: 30330057-30332096	126989	10: 26820350-32294189	MEDAG	ALOX5AP
lincRNA.18895	3: 38182529-38199498	126987	3: 37167143-43070322	PCBP1	ASPRV1
ilncRNA.2931	10: 28848713-28850535	126989	10: 26820350-32294189	N4BP2L1	N4BP2L1

* Chromosome: Start-End.

## Discussion

Owing to the key roles of lncRNAs in many important biological processes (revewied in (Weikard *et al.* 2017; Kopp and Mendell 2018)), they are currently of particular interest. The rapid development of high throughput sequencing methods have led to the discovery of thousands of lncRNAs in recent years. While many lncRNAs have been identified in different organisms such as human and mouse, systematic prediction and regulatory roles of lncRNAs has been poorly studied in domestic animals, particularly in sheep (Bakhtiarizadeh *et al.* 2016). For example, the NONCODE database is an integrated knowledge database dedicated to ncRNAs in 17 species, including human, mouse, cow, rat, chicken and pig (Zhao *et al.* 2016). However, information on sheep lncRNAs in this database is lacking. Also, only five sheep lncRNAs (Xist, MEG3, MEG9, antiPeg11 and Rian) can be found in the other database, lncRNAdb (Quek *et al.* 2015). To our best knowledge, there is no study based on RNA sequencing method regarding the mRNAs and lncRNAs expression profiles in the fat-tail of Iranian sheep breeds. In the present study, a computational framework was used to systematically identify the novel lncRNA and their potential regulatory functions in sheep fat-tail development using RNA-Seq technology.

The results of the cluster analysis based on RNA-Seq data revealed that the same sheep breeds (for example Zel breed) exhibit a greater similarity than the other breed (Lori-Bakhtiari breed). However, a little variation was observed in each breed, which can be attributed to a low level of individual variation. Owing to the rigorous filtering criteria, 424 novel lncRNA transcripts including 358 lincRNAs and 66 ilncRNAs were identified. Similar to mRNAs, novel lincRNAs showed a relatively uniform distribution on the genome. The novel lncRNAs that were discovered in current study expanded the available catalog of lncRNAs in the sheep genome. Our results implied that the identified novel lncRNAs shared many features with previously reported mammalian lncRNAs (Palmieri 2014; Bakhtiarizadeh *et al.* 2016). These findings indicated the reliability of the pipeline that was used for lncRNA identification and the results can provide a good foundation for further analysis. Comparison with known lncRNAs (Bakhtiarizadeh *et al.* 2016; Bush *et al.* 2018) showed that only 23% of all the novel lncRNAs transcripts were mapped, which can be attributed to tissue-specific expression of lncRNAs (Bakhtiarizadeh *et al.* 2016). To better understand the lncRNAs from evolutionary point of view, a comprehensive conservation and synteny analysis was performed. Generally, the results indicated that lncRNAs had modest to low conservation among these species, which is probably a consequence of the degree of genetic closeness of the species being compared. Moreover, low conservation can be attributed to the restrictive patterns during lncRNA evolution. Synteny and conservation analysis of the novel lncRNAs reinforced the hypothesis that synteny of lncRNAs are more conserved than their cross-species sequence conservation, which is in line with our earlier study (Bakhtiarizadeh *et al.* 2016) as well as previous studies (Ulitsky and Bartel 2013; Liu *et al.* 2017).

One of the well-known functions of lncRNAs is regulation of gene expression as they can directly regulate RNA polymerase II (Zhang *et al.* 2018) or promote the phosphorylation of TFs and regulate their DNA-binding activity (Sadallah *et al.* 2011). However, the mechanism underlying the transcriptional regulation by lncRNAs has not been yet understood. Some studies showed that lincRNAs are preferentially located in close proximity to the mRNA genes that they regulate (*cis*-acting) (Jandura and Krause 2017). Therefore, functions of the novel lincRNAs can be inferred by their physical position to mRNAs (genes neighboring lincRNAs). Accordingly, *cis*-prediction analysis showed that there were 12 mRNAs close to the lincRNAs with less than 100 kb distance. It is worth noting that functional enrichment analysis of the neighboring mRNAs revealed that some of the enriched terms were related to the lipid metabolism. Therefore, based on these results, lincRNAs may play an important role in fatty acid biosynthesis, pyruvate and propanoate metabolism and protein digestion and absorption. One of these target genes was ACACA, which is located less than three kb from its potential regulator (lincRNA.3473). Interestingly, the important role of this gene in lipid metabolism is documented in previous studies. ACACA encoding acetyl-CoA carboxylase-α and is a key regulatory enzyme of fatty acid synthesis, catalyzing the carboxylation of acetyl-CoA to malonyl-CoA (Cronan and Waldrop 2002). The different effects of this gene on milk fat content in sheep (Moioli *et al.* 2013; Di Gerlando *et al.* 2017) and cattle (Matsumoto *et al.* 2012), intramuscular fat percentage in bovine skeletal muscle (De Jager *et al.* 2013) and fatty acid composition of meat in pig (Gallardo *et al.* 2009) have been reported. There also, down-regulation of the acetyl CoA metabolic network related genes (including ACACA) in obese individuals with type 2 diabetes compared to individuals with normal glucose tolerance has been demonstrated (Dharuri *et al.* 2014). In this study, ACACA was differentially down-regulated in Lori-Bakhtiari breed and showed the different expression trend with its nearby lincRNA (lincRNA.3473). Interestingly, lincRNA.3473 showed the same synteny with a lncRNA in human, as upstream and downstream genes were ACACA and C17 (or f78). These findings indicated that there might be a negative regulatory relationship between lincRNA.3473 and ACACA and points to an important role in fat deposition. However, further studies are required to elucidate the precise mechanism of action. The other interesting target gene was TSHZ1 that was predicted to be targeted by lincRNA.16164. TSHZ1 is a TF and a member of the teashirt-type zinc-finger protein family, which encodes putative zinc finger TFs (Caubit *et al.* 2000). Previous study reported that the changes in expression of TSHZ1 is correlated with body weight and lipid metabolism in obese individuals (S.Z. *et al.* 2011). These findings implied that the molecular mechanisms underlying the fat deposition in tail of sheep can be controlled by the interactions occurring in a complex network of lncRNAs and mRNAs. In this respect, they may act directly or indirectly on specific TFs through *cis*-acting effects. Collectively, these lincRNAs and their targets are postulated to be potential candidates associated with fat accumulation in tail of Lori-Bakhtiari breed.

One of the other widely used methods that helps to predict the targets of lncRNAs is co-expression analysis through detecting mRNAs with similar expression pattern. This approach enables lncRNAs to regulate protein coding genes distant from their transcription sites. Co-expression analysis revealed that most of lncRNA–mRNA pairs showed the same trend and only four negative significant correlations were found among lincRNAs and mRNAs. Of these, 44 and one target genes of lincRNAs and ilncRNAs were TFs, respectively, which indicates that there is a potential complex network regulating fat-tail development. Furthermore, nine genes that were negatively correlated with lincRNAs were found to be related to calcium signaling pathway. Interestingly, it is well documented that calcium signaling is an important modulator of lipid metabolism. Previous study demonstrated that increasing intracellular calcium in human adipocytes promote energy storage and accumulation of fat content by stimulating *de novo* lipogenesis and inhibiting lipolysis (Xue *et al.* 2001). Moreover, a previous study in pig reported that fat deposition is regulated by calcium signaling pathway (Yu *et al.* 2017). Six of the nine genes were targeted by lincRNA.13110, which may contribute to the fat-tail content differences between the two breeds through regulating their target genes. This hypothesis would be more plausible, since lincRNA.13110 is located in four different QTL regions related to lipid metabolism. In this regard, lincRNA.1974 and lincRNA.23034 were also in QTL regions associated with lipid metabolism, which can be considered as other putative regulators in fat-tail development (Supplementary File S8).

After identifying the co-expressed lncRNAs and mRNAs, integrated network was constructed by combining co-expressed genes and PPI to explore the functional relatedness and potential regulatory relationships among these genes. Finally, three significant modules were considered in the integrated network. Results of the functional enrichment analysis showed that all the modules were significantly associated with biological process terms and KEGG pathways, which were related to lipid metabolism. The importance of insulin signaling pathway in lipid metabolism is well known (Saltiel and Kahn 2001). In this context, enrichment of pathways related with the synthesis of fatty acid and insulin signaling is reported in pigs with divergent phenotypes for fatness traits (Cánovas *et al.* 2010). In this study, regulation of insulin secretion was significant in orange module and two important genes related to this term were ISL1 and FOXA2, which were regulated by lincRNA.2330 and lincRNA.13110, respectively. ISL1 encodes the insulin gene enhancer protein ISL1 (a transcription factor of the LIM homeodomain family), which was initially identified as a regulator of insulin expression (Karlsson *et al.* 1990). Forkhead box protein A2 (Foxa2) is a positive regulator of fatty acid oxidation and an insulin-regulated transcription factor that controls genes relevant to glucose and lipid metabolism (Wolfrum *et al.* 2004). Interestingly, genes related to lipid metabolism and calcium signaling pathways were significantly enriched in purple and blue modules (Supplementary File S7). For example, some of the related genes to lipid metabolism were putative targets for lincRNA.2330 and lincRNA.13110. In the regulatory network shown in Figure 5, both of these lincRNAs were predicted to be the major transcriptional regulators. The results suggested that these lncRNAs may act *in trans* and affect the expression of genes involved in the fat-tail development. Hence, the function of these lincRNAs could be closely related to lipid metabolism as well as fat-tail development, due to their co-expressed targeted mRNAs affecting lipid metabolism.

In the present study, an integrated analysis with the QTL related to lipid metabolism further suggested 100 lincRNAs and 16 ilncRNAs as putative lncRNA candidates in fat-tail development. Two of 100 lincRNAs, lincRNA.12819 and lincRNA.16164, were differentially expressed and their cis target genes were LGSN and TSHZ1, respectively. As discussed above, TSHZ1 is linked to body weight and lipid metabolism in obese individuals (S.Z. *et al.* 2011). In addition, six lincRNAs including lincRNA.6399, lincRNA.17595, lincRNA.26756 lincRNA.2930, lincRNA.2940 and lincRNA.18895 overlapping with QTL associated to “Tail fat deposition” were found, which their cis target genes were related to lipid metabolism. Of these, three lincRNAs including lincRNA.6399, lincRNA.17595 and lincRNA.26756 were localized in QTL regions with interval less than 1 Mb. In the present study, ALOX5AP was predicted as cis target gene of lincRNA.2940. It is worth nothing that the high expression of this gene in adipose tissue and its relevance to body weight has been documented in earlier studies (Kaaman *et al.* 2006; Kaewsutthi *et al.* 2016). The closest protein coding gene to lincRNA.6399 was ITCH. Interestingly, ITCH has been reported as a direct regulator of lipid metabolism in the liver (Stöhr *et al.* 2015). Furthermore, in mice fed a high fat diet, ITCH knockdown improved triglyceride metabolism (Marino *et al.* 2014). The other gene associated with lipid metabolism was TOMM20 that was identified as cis target of lincRNA.17595. A previous study highlighted importance of this gene in metabolic syndrome related lipid alterations (de Toro-Martín *et al.* 2016). lincRNAs that were located in QTL regions related to “Tail fat deposition”, compared with other lincRNAs, are more likely to be truly associated with the fat-tail development. Cis target genes of these lincRNAs were relevant to lipid metabolism. Therefore, these six lincRNAs can be suggested as important candidate lincRNAs in fat deposition in sheep and can provide new insights into the mechanisms behind the fat-tail differences between two breeds. Although our findings require further experimental validation, suggested lncRNAs might regulate lipid metabolism to participate in the regulation of fat-tail development. The present data can potentially help us for further understanding the putative mechanisms involved in fat deposition in sheep fat-tail.

## Conclusion

Here, an attempt was done to identify the novel lncRNAs and their putative functions in fat-tail development in Iranian native sheep breeds. Functional analysis of the putative corresponding targets showed that they were enriched in various functional categories, including lipid metabolism related processes. Identification of the differentially expressed lncRNAs that were neighboring mRNAs related to lipid metabolism yielded novel insights into the regulatory mechanisms of lncRNAs. Additionally, co-expression analysis revealed the putative functions of the identified lncRNAs. A network of regulatory interactions constructed and three significant modules were found, which were related to lipid metabolism. Furthermore, several lincRNAs were found in the QTL regions related to lipid metabolism traits. By finding target genes with known functions in fat metabolism, insulin signaling or calcium signaling pathway, it is speculated that novel lncRNAs may function through neighboring or co-expressed target genes, which might contribute to fat-tail development by regulating these protein coding genes. Our results not only improved the annotation of the sheep genome by reporting the novel lncRNAs, but also provided new insights into the function of lncRNAs in sheep fat-tail development.
